# Sarcoma Immunotherapy: Past Approaches and Future Directions

**DOI:** 10.1155/2014/391967

**Published:** 2014-03-20

**Authors:** S. P. D'Angelo, W. D. Tap, G. K. Schwartz, R. D. Carvajal

**Affiliations:** ^1^Department of Medicine/Melanoma-Sarcoma Oncology Service, Memorial Sloan Kettering Cancer Center, 300 East 66th Street, New York, NY 10065, USA; ^2^Weill Cornell Medical College, 1300 York Avenue, New York, NY 10065, USA

## Abstract

Sarcomas are heterogeneous malignant tumors of mesenchymal origin characterized by more than 100 distinct subtypes. Unfortunately, 25–50% of patients treated with initial curative intent will develop metastatic disease. In the metastatic setting, chemotherapy rarely leads to complete and durable responses; therefore, there is a dire need for more effective therapies. Exploring immunotherapeutic strategies may be warranted. In the past, agents that stimulate the immune system such as interferon and interleukin-2 have been explored and there has been evidence of some clinical activity in selected patients. In addition, many cancer vaccines have been explored with suggestion of benefit in some patients. Building on the advancements made in other solid tumors as well as a better understanding of cancer immunology provides hope for the development of new and exciting therapies in the treatment of sarcoma. There remains promise with immunologic checkpoint blockade antibodies. Further, building on the success of autologous cell transfer in hematologic malignancies, designing chimeric antigen receptors that target antigens that are over-expressed in sarcoma provides a great deal of optimism. Exploring these avenues has the potential to make immunotherapy a real therapeutic option in this orphan disease.

## 1. Introduction/Overview

Sarcomas are a group of heterogenous malignant tumors of mesenchymal origin characterized by more than 100 distinct subtypes. Approximately 13,000 cases of soft tissue and bone sarcomas are diagnosed annually in the US [1]. Surgery, followed by adjuvant radiation for larger tumors, is the mainstay of treatment [2]. Perioperative chemotherapy is used in specific subtypes such as rhabdomyosarcoma, osteosarcoma, and Ewing's sarcoma [2]. Dependent upon initial stage and subtype, 25–50% of patients develop recurrent and/or metastatic disease [3, 4]. Complete responses to chemotherapy for metastatic sarcoma are rare and the median survival is 10–15 months [5, 6]. The development of novel and effective therapies is desperately needed for the treatment of sarcoma.

The immune system is critical in cancer control and progression, and appropriate modulation of the immune system may provide an effective therapeutic option for sarcoma. Thus far, however, no effective immunological therapy for sarcoma has been identified. Nevertheless, building on the progress made in other solid tumors, as well as the expanding understanding of cancer immunology, provides optimism for the development of new immunologic therapies for sarcoma therapies.

Herein, we provide a review of previously investigated immunological therapies for sarcoma and discuss promising future directions. Previously investigated immunotherapies include interferon, interleukin-2, liposomal-muramyl tripeptide phosphatidylethanolamine, and vaccines. Promising future directions for the development of effective immunotherapies include immunologic checkpoint blockade with the targeting of the cytotoxic T-lymphocyte associated protein-4 (CTLA-4) and the programmed cell death protein 1 (PD-1) axis, as well as therapies such as adoptive cell transfer.

### 1.1. History of Immunotherapy in Sarcoma

Immunotherapeutic strategies may be a promising approach to this disease. The role of the immune system as a mechanism of cancer therapy was first observed in sarcoma patients. Dating back to 1866, Wilhelm Busch in Germany observed tumor regressions in patients with sarcoma after postoperative wound infections [7]. Coley described a dramatic response in a patient with small cell sarcoma after an erysipelas infection, suggesting that that the body's response to infection also had potential antitumor effects [8]. He attempted to test this theory by injecting patients with heat-inactivated bacteria to promote an immune response [8]. He treated a patient with recurrent head and neck sarcoma with local injections of streptococcal broth cultures and noted a near complete response which lasted close to eight years [9]. The data generated by Coley was not reproducible given its inconsistent nature and, ultimately, the American Cancer Society refuted the role of Coley's toxin as an effective treatment [10].

The observation that the development of sarcoma is more common in patients that are immunosuppressed also supports the relevance of the immune system in this disease [11]. The development of sarcomas has been described in allograft transplant recipients. In a study of 8191 transplant patients, 8724 malignancies occurred and 7.4% of them were sarcomas [12]. While a majority of patients developed Kaposi sarcoma, 1.7% of patients developed other sarcomas including malignant fibrous histiocytoma (MFH), leiomyosarcoma (LMS), fibrosarcoma, rhabdomyosarcoma, hemangiosarcoma, and undifferentiated sarcoma which is nearly tripled compared to an incidence of 0.5% in the general population [12].

### 1.2. Innate and Adaptive Immunity in the Nonmalignant State

The immune system is comprised of the innate and adaptive arms [13]. The innate immune system includes dendritic cells (DC), natural killer cells (NK), macrophages, neutrophils, basophils, eosinophils, and mast cells [13]. Innate immune cells serve as the initial defense against foreign antigens. Once activated, macrophages and mast cells release cytokines that engage additional immune cells and initiate an inflammatory response [13]. Dendritic cells can serve as antigen presenting cells (APC). They uptake foreign antigens and present them to the adaptive immune cells, providing interaction between the 2 arms of the immune system. Natural killer cells can both activate DC and eliminate immature DC. Through their interaction with the DC, NK cells can also provide interplay between the innate and adaptive immune system.

The adaptive immune system includes B lymphocytes, CD4^+^ helper T lymphocytes, and CD8^+^ cytotoxic T lymphocytes (CTLs) and requires formal presentation by APC for its activation. The adaptive immune system generates T/B cell lymphocytes that are antigen specific. These pathways work together to eliminate invading pathogens and damaged cells [13].

### 1.3. Immune System and Tumorigenesis

**Figure 1:**
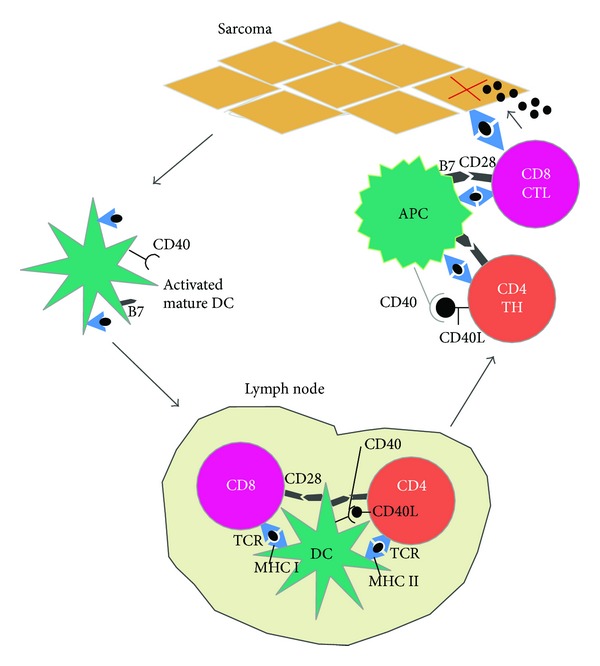
Preventing tumorigenesis in sarcoma. The adaptive immune response initiates with presentation of antigens by DC. DC migrates to the lymph node. Antigens are presented to CD4^+^ and CD8^+^ T cells through MHC class I and II, respectively, and costimulatory molecules such as B7 bind to CD28 leading to activation of the lymphocytes. Once stimulated, these lymphocytes are now effector cells with the ability to migrate to the tissue and initiate an immune response against the developing sarcoma (abbreviations: CTL, cytotoxic T lymphocyte; DC, dendritic cell; TH, T helper lymphocyte). Reference: adapted from [25].

Antitumor immunity requires antigen presentation by DC, production of T cell responses, and overcoming immunosuppression at the tumor bed [14]. T cell activation requires dual signaling [15, 16]. The binding of the T cell receptor to antigens presented by antigen presenting cells via major histocompatibility classes (MHC) I and II is the first required signal. The second signal is generated when the B7 ligand binds to CD28, a costimulatory receptor. This signaling leads to T cell proliferation, cytokine release, and upregulation of the immune response. After DC present tumor antigens on the MHC I or II, they migrate to nearby lymph nodes [14]. If the appropriate maturation signal is present, the DC elicit effector T cell response in the lymph node. Further, the interaction of other T cell stimulation molecules such as CD28 or OX40 with CD80/86 or OX40L will promote a protective T cell response (Figure 1).

Tumor antagonizing immune cells such as CTLs and NK cells are believed to play a role in eradication of tumors [17]. It has been demonstrated that mice with impaired function of CD8^+^ CTLs, CD4^+^ Th1 helper T cells, or NK cells have increased incidence of tumors [18, 19]. As such, individuals that are immunocompromised have increased incidence of certain kinds of cancers [20].

Lack of the immunogenic maturation stimuli will lead to T-cell depletion and production of regulatory T cells (Tregs) [14]. Further, interaction of CTLA-4 with CD80/86 or PD-1 with PD-L1/PD-L2 will also suppress the T cell response. CTLA-4 competes with B7 for CD28 binding. Since, CTLA-4 has higher affinity for the CD28 receptor, the T cell response is downregulated. CTLA-4 is thus a negative regulator of T cell responses that prevents autoimmunity and allows tolerance to self-antigens [15].

**Figure 2:**
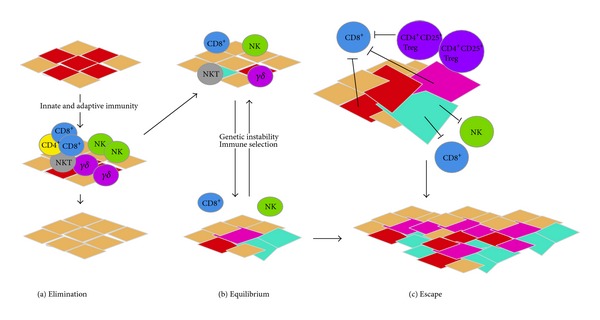
Immunoediting. The 3 phases of immunoediting include elimination, equilibrium and escape. (a)* Elimination*. Cancer cells are transformed (red) but are actively destroyed by the cells of the immune system. (b)* Equilibrium*. Cancer cells continue to transform (red and teal). Immune system cannot completely remove the transformed cells but controls their growth and there is a dynamic equilibrium that keeps the tumor in check. (c)* Escape*. Cancer cells continue to grow and transform (red, teal, and pink). These cells now grow unchecked and exhibit immunosuppressive mechanisms (CD4^+^CD25^+^ Treg) which ultimately lead to progressively growing tumors. Abbreviations: CD4^+^, CD8^+^, CD4^+^CD25^+^ Treg, *γδ* and NKT cells are all types of T cells and NK cells are natural killer cells. Reference: figure modified from [21].

PD-1 is a member of the CD28 family of T-cell costimulatory receptors that also includes CD28, CTLA-4, inducible T cell costimulator, and B and T lymphocyte attenuators [21]. PD-1 is expressed on activated T cells, B cells, and myeloid cells [22]. There are 2 ligands, programmed cell death ligand (PD-L1 and PD-L2) that are specific for PD-1. Once they bind to PD-1, downregulation of T-cell activation occurs [23, 24]. If this interaction is interrupted, the checkpoint is turned off and antitumor T-cell activation may be enhanced (Figure 2).

Ultimately, antigen primed T cells, B cells, and NK cells will exit the lymph node and enter the tumor bed. The tumor microenvironment can have additional defense mechanism which can also oppose the tumor response. Therefore, the tumor-associated inflammatory response can also have a paradoxical effect leading to tumor growth and progression [17]. Tumor promoting immune cells such as macrophage subtypes, mast cells, neutrophils, and certain T and B lymphocytes can prevent immune destruction of the tumor by secreting immunosuppressive factors. Suppression of the tumor immune response has been termed immune-evasion; this is emerging as the seventh hallmark of cancer [17].

### 1.4. Immunoediting

**Figure 3:**
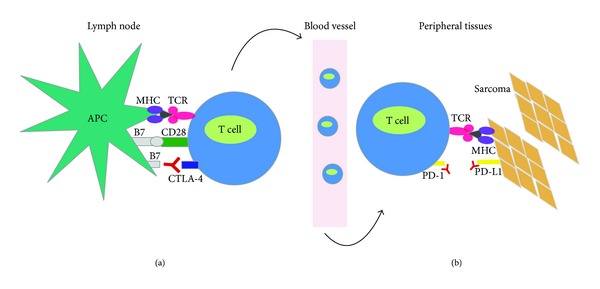
Mechanism of action of CTLA-4 and PDL1 blockade. (a) Activation of T cell requires interaction of MHC bearing tumor antigen with the TCR and interaction of the costimulatory molecule B7 with CD28. CTLA-4 is a negative regulator of the immune response that competes with CD28 binding with B7. Ipilimumab is a monoclonal antibody that binds CTLA-4 and promotes continued T cell activation. (b) The role of the PD-1 receptor is more significant in the peripheral tissue, once T cell activation has already occurred. After antigen exposure, PD-1 receptor is expressed on the T cells. When the PD-1 receptor interacts with its ligands PD-L1 and PD-L2, there is negative regulation of T cells in the tumor microenvironment. Blocking PD-1 or PD-L1 leads to activation of T cells. Reference: figure modified from [57].

Immunoediting has been demonstrated preclinically in murine models of sarcoma [26]. Cellular immunity as a mechanism of protection from cancer was first described as the concept of immunosurveillance [27]. Immunosurveillance may in fact be the first phase of immunoediting. The immunoediting paradigm is a larger process describing how an individual is protected from cancer growth and develops tumor immunogenicity [27–30]. Immunoediting includes 3 phases: elimination, equilibrium, and escape [31, 32] (Figure 3). In the elimination phase, the immune system is capable of recognizing and destroying cancer cells. Examples include (1) lymphocytic infiltration of the tumor, which has been demonstrated in sarcomas such as Ewing's sarcoma and GIST [33–35] and (2) spontaneous regression of primary tumors which have been seen in desmoid tumors and osteosarcomas [36, 37]. A case report described a 15-year-old girl that presented with a large 6 cm × 8 cm desmoid tumor in the left iliac fossa. The patient was considered inoperable given the extent of disease and involvement of local structures. Ultimately, after 5 years, the tumor began to regress and, by 10 years, it was no longer palpable [36]. In another example, a 57-year-old woman underwent surgical resection of a proximal thigh extraskeletal osteosarcoma [37]. The pathology specimen noted tumor cells that had been replaced by fibrocollagenous tissue with lymphocytic infiltration. The prognostic implications of lymphocytes in soft tissue sarcomas have been reported [38, 39]. Tissue microarrays were constructed and immunohistochemistry was used to evaluate multiple white blood cells that play a role in the immune system these including CD3^+^ (T cell coreceptor), CD4^+^, CD8^+^, CD20^+^ (expressed on B cell surface, which plays a role in B cell activation), and CD45^+^ lymphocytes (lymphocyte common antigen, which plays a role in T cell activation) in tumors [39]. CD20^+^ lymphocytes in resected soft tissue sarcoma were independent positive prognostic factors associated with improved disease specific survival [39].

In the equilibrium phase, cancer cells maintain a balance with the immune system where they are able to avoid immune-mediated destruction but are not able to progress [40, 41].

In the escape phase, cancer cells grow and metastasize due to loss of control by the immune system [32]. The loss of MHC I on sarcoma cells is an example of the escape phenomenon [42]. MHC 1 loss was demonstrated in 46/72 (62%) of bone and soft tissue sarcomas. In the 21 osteosarcoma patients, expression of MHC I was associated with improved overall and event-free survival [42].

## 2. Previously Investigated Immunotherapies

### 2.1. Cytokines

Stimulation of the immune system has been evaluated with cytokines such as interleukin-2 (IL-2) and interferon (IFN). Cytokines regulate the function of the immune system and are involved in antigen presentation as well as T cell activity [43].

IL-2 stimulates and upregulates T and NK cells and mediates lymphocyte proliferation [44–46]. IL-2, which typically binds to the type I cytokine receptors [43], has the ability to activate a population of lymphocytes into lymphokine-activated killer cells (LAK) [47]. LAK cells are lymphocytes that have the potential to eradicate tumor cells regardless of histocompatibility expression [48–50]. It has been demonstrated that lymphocytes that are stimulated with IL-2 can lyse malignant cells [51]. Responses to IL-2 have been described in ovarian, nonsmall cell lung, breast, melanoma, and renal cell cancers [51]. High dose infusional IL-2 is FDA approved for the treatment of metastatic melanoma and renal cell carcinoma [52, 53]. Limitations of this therapy include significant treatment associated toxicity.

In a small study of high dose IL-2 used in conjunction with LAK cells, none of the six sarcoma patients treated responded [54]. A more recent study of high dose IL-2 in 10 heavily pretreated pediatric patients with multiple malignancies included 4 patients with osteosarcoma and 2 patients with Ewing's sarcoma [51]. Of the four osteosarcoma patients, two achieved complete responses that were durable, with a median followup of 28 months while the other two had progressive disease. Both patients with Ewing's sarcoma had progressive disease. The responses observed with IL-2 suggest that this agent may have efficacy in a subset of patients with sarcoma [51]. The future of IL-2 as a single agent for the treatment of sarcoma patients is not known; there is, however, a study combining IL-2 with vaccine therapy (NCT00101309). The rationale is to potentially improve on the efficacy of either agent alone by enhancing the immune response.

### 2.2. Interferon

Interferons are a family of molecules which bind to either type I or type II IFN receptors, which are subsets of the type II cytokine receptors [43, 55]. IFN*α* and IFN*β* both activate the type I IFN receptors; IFN*γ* binds distinctly to the type II IFN receptors [55]. Since they bind to the same receptors, both IFN*α* and IFN*β* likely have similar biological effects; however the actual mechanism of antitumor activity is not clear [55]. IFN*α* and IFN*β* are capable of activating immune cells and increasing antigen presentation to T lymphocytes [55]. There are many different forms of IFN*α*, including IFN*α*-2a, IFN*α*-2b, and IFN*α*-2c all which vary by a few amino acids [55]. IFN*α* is approved in the adjuvant setting for high-risk melanoma. In a cooperative group study, patients with stage II or III melanoma received 20 million units/m2/day IV 5 days per week, followed by 10 million units/m2/day SQ 3x per week for 48 weeks. This study demonstrated an initial survival benefit that subsequently was lost with longer followup [56].

There have been multiple studies with interferon in sarcoma. A case report of patients with metastatic disease described two of three patients with osteosarcoma who received interferon and achieved partial responses [58]. A phase II study demonstrated that three of twenty patients with metastatic bone sarcomas (2 with osteosarcoma and 1 with malignant fibrous histiocytoma) received recombinant interferon *α*-2a and achieved short lasting partial responses [59]. In the adjuvant setting, there have been many studies investigating the role of adjuvant interferon. While some studies appeared promising, none reached statistical significance. There is an on-going randomized trial of the European and American Osteosarcoma Study group for patients with resectable osteosarcoma with favorable responses to preoperative chemotherapy. This study randomizes patients to two different combination chemotherapy regimens with or without PEG-INT *α*-2b (NCT00134030) (Table 1).

### 2.3. MTP

Liposomal-muramyl  tripeptide phosphatidyl-ethanolamine (L-MTP) has also been investigated to potentially stimulate the immune system. MTP is a synthetic analogue of a bacterial cell wall that is capable of activating monocytes and macrophages [60]. MTP can result in inflammation, release of antimicrobial peptides, fever, DC recruitment, and potential tumor cell death [60]. The rationale for using MTP in oncology is to trigger an inflammatory response to eradicate micrometastatic disease [60].

The Children's Oncology Group Intergroup-0133 was a 4-arm study in which patients with osteosarcoma without clinically detectable metastatic disease were double randomized at the time of study entry. The first randomization was to receive adjuvant cisplatin, doxorubicin, and high-dose methotrexate with or without ifosfamide. The second randomization was to receive either 3-drug or 4-drug chemotherapy alone or 3-drug or 4-drug chemotherapy plus liposomal-MTP. In a pooled analysis the study demonstrated that the addition of ifosfamide to cisplatin, doxorubicin, and high-dose methotrexate did not improve either event-free or overall survival. The authors concluded that there was improved survival (both event-free and overall) in those patients that received chemotherapy with L-MTP with an increase in the 5-year overall survival rate from 70% to 78% (*P* = 0.03; relative risk, 0.73) [61]. In patients who presented with metastases, there was also a benefit in event-free and overall survival although the analysis was not powered to support a statistically significant benefit [62]. L-MTP is approved for use in the European Union, Mexico, Turkey, and Israel. It is not FDA approved. A compassionate study of L-MTP for patients with high-risk osteosarcoma was completed in December of 2012 which also demonstrated a survival advantage for the patients who received L-MTP [63].

### 2.4. Vaccines

There have been multiple clinical trials investigating vaccines targeting whole cells, lysates, proteins, and peptides in patients with sarcoma. Vaccines can be combined with costimulatory adjuvants as well as immunostimulants such as GM-CSF or IL-2 to potentially enhance the immune response. The goal of vaccination is to expose patients to tumor antigens with hope of inducing an antitumor immune response with the generation of tumor specific antibodies or T cells that ultimately translates to clinical benefit [64]. Many studies have yielded disappointing results, although there were some patients that derived benefit (Table 2).

Currently, there is a phase II trial of a trivalent peptide vaccine to the gangliosides GD2, GD3, and GM2 in patients with sarcoma that have had solitary metastases excised (NCT00597272). This study has been closed to accrual and results are pending. Perhaps the ideal study population for vaccines includes postoperative patients to minimize micrometastases.

## 3. Immunologic Checkpoint Blockade

### 3.1. CTLA-4 Blockade

Ipilimumab is a human monoclonal antibody that binds CTLA-4 that is FDA approved for the treatment of metastatic melanoma [76]. In a small phase II study, patients with synovial sarcoma were treated with ipilimumab 3 mg/kg every 3 weeks and restaged after 3 cycles [77]. The primary endpoint of the study was RECIST 1.0 response rate. Secondary endpoints included determination of the clinical benefit rate and evaluation of NY-ESO-1 specific immunity. Four patients completed 3 doses of ipilimumab, while 2 patients each received 1 and 2 doses due to clinical or radiologic progression. There were no documented responses, and the time to progression ranged from 0.47 months to 2.1 months. There was no evidence of serologic or delayed type hypersensitivity to NY-ESO-1.

Although ipilimumab has demonstrated an improvement in overall survival in metastatic melanoma, the response rate is only 10–20% [78]. Clinical responses can be delayed and some patients do not demonstrate disease regression or stabilization for many weeks after therapy is complete. There are patients who have initial progressive disease and subsequent disease stabilization. Taking into account what we learned from melanoma, selecting progression-free survival or overall survival as primary endpoints may lead to a better designed study. Incorporation of the immune-related response criteria (irRC) may be prudent. With standard cytotoxic chemotherapy, typical patterns can include an increase, decrease, or no change in tumor burden which can be effectively interpreted with the response evaluation criteria in solid tumors (RECIST) [79]. With immunotherapy, patients can sometimes have a response after an increase in tumor burden or a response in the presence of new lesions. Per RECIST, this would qualify as disease progression. Progression as judged by tumor size may be deceptive as an increase in immune cell infiltration into the tumor may appear on a radiographic study as an increase in tumor size. Immune-related response criteria are a set of novel response criteria designed to capture these unique response patterns. The irRC were devised using the data from the phase II clinical trials of patients with metastatic melanoma treated with ipilimumab [80]. With the irRC, progressive disease is defined as total disease growth up to 25% from baseline or total disease burden (new lesions plus target) greater than 25%.

Therefore, it may be best to consider ipilimumab in the early metastatic setting, when patients have less disease burden. A patient with rapidly progressive disease may not be the best candidate. If interval imaging studies are performed, they should be interpreted with caution, as disease progression early on may not necessarily translate to lack of efficacy.

### 3.2. Enhancing the Activity of CTLA-4 Blockade

Therapies that can induce cell death such as radiation therapy, traditional chemotherapies, and targeted therapies can result in the release of antigens. These antigens may serve as priming events for immune specific responses mediated by CTLA-4 blockade [81, 82].

Targeted therapies such as tyrosine kinase inhibitors (TKIs) have “off-target” effects on the immune system and suppressing and stimulating effects on immune cells, such as CD4^+^ and CD8^+^ T cells [83, 84], NK cells [85], and DC [86]. These immune effects have been associated with improved preclinical [84] and clinical outcomes [85, 87].

The effective treatment of human GIST tumors with imatinib is associated with an increased intratumoral CD8^+^ effector T cells (Teff)/regulatory T cell (Treg) ratio [88]. Regulatory T cells contribute to decreased immune responses to tumors [89–92]. Tumors that are pathologically resistant to imatinib are associated with a lower Teff/Treg ratio which has been correlated to the level of indolamine 2, 3-dioxygenase (IDO) [88]. IDO is a heme-containing enzyme that catalyzes the oxidative breakdown of the essential amino acid tryptophan, via the kynurenine pathway [93]. IDO has been shown to inhibit T-cell proliferation and blockade of cell cycle progression by tryptophan depletion. Consideration of IDO blockade warrants further study, either alone or in combination with other immunotherapeutic strategies.

In a KIT-mutant GIST mouse model there has been augmentation of the antitumor effect of KIT-targeting with CTLA-4 blockade leading to improved, more durable responses [88]. There is a phase Ib/II study of ipilimumab with dasatinib for patients with soft tissue sarcoma with an expansion of GIST (NCT01643278).

### 3.3. PD-1 Blockade

The PD-1 receptor is another promising potential immunological target. In a phase I study that enrolled and treated 296 patients with nivolumab, an antibody to PD-1, response rates were 18%, 28%, and 27% in patients with nonsmall cell lung cancer (NSCLC), melanoma, and renal cell carcinoma (RCC), respectively [94]. There was suggestion that PD-L1 expression by immunohistochemistry may correlate with clinical activity of PD-1 blockade. Pretreatment expression of PD-L1 was performed in 42 patients. There were 18 patients that lacked expression and all of them did not have any evidence of benefit to the drug. Of the 25 patients with PD-L1 expression, 9/25 (36%) had an objective response, *P* = 0.006 [94]. A phase I study of nivolumab and ipilimumab in patients with advanced melanoma demonstrated impressive objective response rates of 40% [95]. Among patients that received combination therapy, responses were seen both in patients with PDL-1 expression (6/13) or those without PDL-1 expression (9/22).

Even though the role of PDL-1 expression as a biomarker remains debatable, evaluation of sarcoma tumor specimens for expression of PDL-1 may provide potential justification for a clinical trial.

## 4. Exploring Surface Antigens and Cancer Testis Antigen

Many sarcomas express specific epitopes due to the cell of origin or as a result of gene products providing targets for immune-mediated therapeutic approaches. Prospective targets include cancer testis antigens that are commonly expressed in many sarcomas [64, 96]. Gangliosides and cancer testis antigens may prove to be potential targets for adoptive T cell transfer as well as for vaccine development.

### 4.1. Gangliosides

Gangliosides are glycosphingolipids containing a lipid component and a carbohydrate chain that are found on the cell surface that are believed to play a role in cell attachment and cell-cell interactions. Several of these gangliosides, including GM2, disialoganglioside (GD2), and GD3, are expressed by tumors such as melanoma [97–100], sarcomas, and neuroblastoma [101–103].

Using immunohistochemistry, gangliosides and protein antigens were explored as potential targets for immunotherapy in sarcomas [100, 104, 105]. Fresh frozen human sarcoma tumor samples were evaluated for GD2 and GD3 (using the purified mAbs 3F8 and R24, resp.) and demonstrated that 93% of the tumors expressed GD2 and 88% expressed GD3 by immunohistochemical staining [99, 106]. Certain histologic types showed a greater extent of expression of GD2 and GD3, including liposarcoma, fibrosarcoma, malignant fibrous histiocytoma, leiomyosarcoma, and spindle cell sarcoma. Monophasic synovial sarcoma, embryonal rhabdomyosarcoma, alveolar soft part sarcoma, and hemangiosarcoma demonstrated less staining.

### 4.2. Cancer Testis Antigens

The cancer testis antigens (CTA) are proteins that are typically found of tumor cells and lack expression on normal tissue [107]. Approximately 20 CTA have been identified [107]. These antigens are typically expressed on germline tissues, placental trophoblasts, as well as specific cancers but most importantly, they can be recognized by CTL [108]. In order to be recognized by CTL, there must be adequate expression of human leukocyte antigen (HLA) class I by tumor cells [109]. Every CTA has epitopes for a minimum of one HLA type, although many of these HLA types remain uncommon [64]. Since class I HLA A*02.01 is found in approximately half the Caucasian population, many immunotherapy trials target HLA A*02.01 associated antigens such as NY-ESO-1, LAGE-1, PRAME, MAGE-A3, MAGE-A4, MAGE-A9, and SSX-2. While exploration of these tumor antigens has historically been focused on tumors with known spontaneous immunogenicity there has been more interest in exploring expression of CTA in sarcoma. Studies have utilized RT-PCR and IHC methodology to explore known CTA expressed in other malignancies [109–112]. Results are demonstrating that CTA are potential targets in sarcoma. Studies to date have shown that antigen expression does vary by sarcoma subtype (Table 3).

## 5. Adoptive Cell Transfer 

Adoptive cell transfer (ACT) involves the transfer of immune cells with antitumor activity. The mechanisms by which this can occur include the expansion and infusion of tumor infiltrating lymphocytes, the use of genetically modified lymphocytes, or the use of chimeric antigen receptors (CAR).

By using gamma retroviruses or lentiviruses, lymphocytes can be genetically modified to encode T cell receptors (TCR) that recognize specific tumor antigens or encode molecules that can enhance their antitumor activity [114]. These lymphocytes then acquire antitumor activity. Historically, these are conventional TCR that have alpha and beta chains that form heterodimers to recognize cancer antigens that are presented on the surface of MHC molecules on tumor cells [114]. T cells genetically engineered to target NY-ESO-1 expressing synovial sarcoma have shown some promise [115]. In a small study, patients with NY-ESO-1 expressing synovial sarcomas were treated with a lymphodepleting chemotherapy regimen consisting of cyclophosphamide and fludarabine, followed by infusions of autologous T lymphocytes designed to recognize a specific NY-ESO-1 antigen. Four of 6 patients with synovial sarcoma had evidence of partial response lasting from 5–18 months. Limitations of conventional TCR are that they are restricted to antigen presentation on specific MHC molecules [114]. In addition, downregulation of class I MHC molecules has been described as a mechanism of avoiding TCR recognition [116].

CAR are composed of the antigen-combining regions of the heavy and light chains of antibodies with T-cell intracellular signaling molecules [117]. CAR provide the opportunity to recognize antigens based on antibody interaction [117]. T cells can express CAR targeting different tumor-associated antigens such as GD2 which is expressed in neuroblastoma, melanoma, and sarcoma [118] or CD19 B cell antigen which expressed in non-Hodgkin lymphomas as well as chronic lymphocytic leukemia [119]. There have been phase I clinical trials using CAR to treat ovarian [120], renal cell carcinoma [121], lymphoma [122], and neuroblastoma [123]. Results have been disappointing, with minimal clinical responses, possibly due to lack of persisting CAR-expressing T cells [124]. The first generation CAR lack costimulatory signals such as CD28, 4-1BB, and OX40 and are only composed of an intracellular signaling domain derived from the TCR CD3-*ξ* chain [125–128]. Second and third generation CAR include a CD28 signaling domain as well as OX40 or 4-1BB, respectively [118, 129, 130].

Presumably, second and third generation CAR should lead to more effective responses and persistence of the CAR. Thus far, there has been exciting data in hematological malignancies showing promise of second generation CAR [131]. The hope is that this success can be translated to similar efficacy in solid tumor malignancies.

## 6. Future Directions

To date, the field of sarcoma immunotherapy has not yet matured to show robust antitumor effects, but there has been suggestion of clinical activity in some patients. Although there have been multiple clinical trials evaluating the role of stimulants of the immune system such as IL-2 and IFN, these agents have failed to improve overall survival [51, 54, 58, 59]. MTP has shown promise demonstrating improvement both in event and overall survival [61–63]. The study design may have limited FDA approval, although this agent is available in the European Union, Mexico, Turkey, and Israel. Nonetheless, these drugs appeared to have offered benefit only for selected patients. There are now improved therapies and the success of these therapies in diseases such as melanoma has offered a better understanding of immunology. Through the melanoma experience, we have learned that more appropriate patient selection can perhaps lead to more successful clinical trials. Melanoma and sarcoma are clearly two distinct malignancies; however, like melanoma, sarcoma does have infiltration of tumor associated lymphocytes, which can provide rationale for immunomodulation [38, 39]. Like patients with melanoma, there have been cases of spontaneous regression in patients with sarcoma [36, 37]. Sarcoma does have evidence of effective immunosurveillance as demonstrated by Coley [8] and Wiemann and Starnes [9]. As noted in patients with GIST, TKIs such as imatinib can have stimulating effects on multiple immune cells [88]. Therefore immunotherapies may be effective in this disease, if the appropriate drugs are used in the appropriate patients. Moving forward, more precise immune modulation, enhancing activity of immunomodulatory agents, and targeting sarcoma specific epitopes may all lead to a more successful approach in treating this disease.

Investigating immunomodulators such as ipilimumab may be promising. Although it was only previously investigated in synovial sarcoma, our knowledge regarding this agent may in fact lead to a better designed trial [77]. Previously, solid tumors such as NSCLC did not appear to be immunogenic tumors. Yet, we have since learned that anti-PD-1 antibodies have led to durable and effective responses in lung cancers [94].

Through small numbers, the data with NY-ESO-1 T cell therapy appeared to be efficacious in some patients, providing hope [115]. Perhaps utilizing a CAR to target an epitope that is overexpressed in sarcoma may broaden applicability of this intervention to other sarcoma subtypes.

The knowledge and current understanding of the immune system can build enthusiasm to create a niche for sarcoma therapy. Moving forward, it is important to model the development of immunological therapies in sarcoma after the successful development of such therapy in other solid tumors. While there may not be any biological parallels amongst these malignancies, a better understanding of antitumor immunity mechanism may be incorporated into designing more rationale clinical trials.

## Figures and Tables

**Table 1:** Adjuvant interferon studies.

Study name	Patients	Type of sarcoma	Type of interferon	Dose/schedule	Outcome
Karolinska Hospital series	After surgical resection, 89 patients	Osteosarcoma	Interferon-*α*	Cohort 1 (70 patients) 3 × 10^6^ IU daily ×1 month, followed by 3×/week, 17 months Cohort 2 (19 patients) 3 × 10^6^ IU daily for 3–5 years	10-year metastatic free survival: 39% (95% CI 29–49%) 10-year sarcoma specific survival: 43% (95% CI 33–54%) [65, 66] Median time to metastasis 8 months (range 1–60 months) *P* value not specified

COSS-80	100 patients after preoperative chemotherapy and surgical resection were randomized +/− interferon	Osteosarcoma	Interferon-*β*	100,000 U/kg for 22 weeks (2 injections weekly ×2 weeks, daily ×4 weeks, weekly ×16 weeks)	30-month continuous disease-free survival: 77% interferon arm versus 73% noninterferon arm [67] Log rank test, *z* = 0.315

EURAMOS 1	715 patients that had good response to preoperative chemotherapy were randomized to postoperative chemotherapy +/− interferon	Osteosarcoma	Pegylated interferon-*α*2b	0.5–1.0 *μ*g/kg/week for 2 years	Primary endpoint: event-free survival (EFS) [68] 77% who received INT were disease free at 3 years compared to 74% Hazard ratio for EFS from adjusted Cox model was 0.82 (95% CI 0.61–1.11; *P* = 0.201) in favor of chemotherapy + interferon

**Table 2:** Vaccine studies in sarcoma.

Vaccine	Sarcoma histology	Number of patients	Immune response	Results
Irradiated autologous tumor cells [69]	Various pediatric	16	Not reported	16.6 m versus 8.2 m survival (skin test responders)

Dendritic cell pulsed with autologous tumor lysate [70]	Various pediatric	10	Not reported	One response in patient with fibrosarcoma

Dendritic cell pulsed with peptides from tumor specific translocation breakpoints and E7 [71]	Metastatic Ewing's family of tumors or alveolar rhabdomyosarcoma	30/52 initiated vaccine after standard therapy	39% with immune response to translocation breakpoint 25% with E7-specific response	5-year OS: 31% for all patients versus 43% for patients initiating immunotherapy

105AD7 (CD55 target) [72]	Osteosarcoma	28 patients within 1–6 months of chemotherapy	20/28 (71%) showed T cell proliferation response in vitro to 105AD7 9/28 (32%) weak antibody response to CD55	2 patients with possible clinical responses, alive and disease free 5.8 and 6.5 years from time of diagnosis

Dendritic vaccine pulsed with synthetic tumor specific peptide [73]	Posttransplant, residual tumor (synovial, Ewing's)	5	DTH response against tumor lysate in 1 patient	1 patient complete response, 77 months

Peptide encompassing SYT-SSX [74]	Synovial	6	Peptide specific CTLs generated in 4 patients	Suppression of tumor progression 1 patient

SYT-SSX derived peptide [75]	Synovial	20	9 showed twofold increase in CTLs in tetramer analysis	1/9 stable disease (received vaccine with peptide alone) 6/12 stable disease (received vaccine with incomplete Freund's adjuvant)

Abbreviations: CTL.

**Table 3:** Cancer testis antigens expressed in sarcoma.

Antigen	Sarcoma type	Total expression
NY-ESO [110, 111, 113]	Synovial Myxoid round cell liposarcoma Uterine leiomyosarcoma Osteosarcoma	80% 100% 50% (3/6) 89% (8/9)

LAGE [109, 112]	Myxoid round cell liposarcoma Nonmyxoid liposarcoma Osteosarcoma	70% 60% 89% (8/9)

PRAME [112]	Synovial sarcoma Nonmyxoid liposarcoma Myxoid liposarcoma	100% (4/4) 100% (4/4) 14% (1/7)

MAGE-A3 [109]	Uterine leiomyosarcoma Nonuterine leiomyosarcoma	67% (4/6) 14% (1/7)
